# Off-treatment durability of antiviral response to nucleoside analogues in patients with chronic hepatitis B

**DOI:** 10.1186/s12876-016-0454-z

**Published:** 2016-03-17

**Authors:** Naruhiko Nagata, Tatehiro Kagawa, Shunji Hirose, Yoshitaka Arase, Kota Tsuruya, Kazuya Anzai, Koichi Shiraishi, Tetsuya Mine

**Affiliations:** Division of Gastroenterology and Hepatology, Department of Internal Medicine, Tokai University School of Medicine, Shimokasuya 143, Isehara, 259-1193 Japan

**Keywords:** Chronic hepatitis B, Nucleoside analogue, Durability, Hepatocellular carcinoma, HBs antigen

## Abstract

**Background:**

Off-treatment durability of nucleoside analogue (NA) therapy in patients with chronic hepatitis B has not been well investigated. In this study we monitored antiviral effect of NA therapy and evaluated off-treatment durability after NA cessation in patients with chronic hepatitis B.

**Patients and methods:**

A total of 94 consecutive patients (39 HBeAg-negative and 55 HBeAg-positive patients) who received NA therapy were followed up for approximately 9 years. We discontinued NA according to the following criteria; undetectable serum HBV-DNA by polymerase chain reaction (PCR) on three separate occasions at least 6 months apart in HBeAg-negative patients (APASL stopping recommendation), and seroconversion from HBeAg-positive to HBeAb-positive and undetectable serum HBV-DNA by PCR for at least 12 months in HBeAg-positive patients.

**Results:**

The cumulative rate of relapse after NA cessation was 48 % and 40 % in HBeAg-negative and -positive patients, respectively. Higher baseline serum alanine aminotransferase level was the only significant predictor for maintaining remission. No patients experienced decompensation after relapse. HBsAg loss occurred at an annual rate of 1.4 % and 0.4 % in HBeAg-negative and -positive patients, respectively. Hepatocellular carcinoma developed at an annual rate of 0.6 % in both HBeAg-negative and -positive patients.

**Conclusions:**

Almost half of the patients did not relapse after cessation of NA therapy in both HBeAg-negative and -positive patients. Therefore, NA therapy could be discontinued with close monitoring if the APASL stopping recommendation is satisfied even in HBeAg-negative patients.

## Background

Nucleoside analogues (NAs) are shown to improve prognosis of patients with chronic hepatitis B. Continuous treatment with lamivudine (LMV) delayed clinical progression defined by hepatic decompensation, hepatocellular carcinoma (HCC), spontaneous bacterial peritonitis, bleeding gastroesophageal varices, or liver disease-related death in patients with chronic hepatitis B and advanced fibrosis or cirrhosis [1]. NAs can suppress hepatic inflammation by inhibiting viral proliferation in most cases, however, this effect is transient. Relapse after NA cessation is frequently observed [2–9]. Alanine aminotransferase (ALT) flare with 10 times higher than normal upper limit was reported in up to 20 % [2, 3] of patients who discontinued NA treatment. These results suggest that NA discontinuation should be decided with caution. On the other hand, considerable number of patients are receiving NA treatment even if they actually no longer need NA. Therefore, making a discrimination of patients who can discontinue NA treatment is important from the viewpoint of health care economics.

Currently there is no clear consensus on when to stop NA treatment. For HBeAg-negative patients, the guidelines from the American Association for the Study of Liver Diseases (AASLD) [10] and the European Association for the Study of the Liver (EASL) [11] recommend long-term NA treatment until HBsAg seroclearance has been achieved. Although HBsAg seroclearance is an ideal goal of NA treatment, it takes place in only a minority of patients (<1 % per year [10]). On the other hand, the Asian-Pacific Association for the Study of the Liver (APASL) guideline [12] suggests that treatment discontinuation can be considered if patients have been treated for at least 2 years with undetectable HBV-DNA documented on three separate occasions 6 months apart, even if HBsAg seroclearance is not achieved. For HBeAg-positive patients, the AASLD, EASL and APASL guidelines recommend that NA treatment can be discontinued when HBeAg seroconversion with undetectable HBV-DNA has been maintained for 6-12 months.

There have been several studies on off-treatment durability of NA’s antiviral effect (see review [13]), however, the results considerably vary by study. Therefore, the accumulation of further data is required.

In this study we monitored antiviral effect of NA therapy and evaluated off-treatment durability after NA cessation according to the APASL stopping recommendation in patients with chronic hepatitis B.

## Methods

We consecutively enrolled the patients who started treatment with LMV or entecavir (ETV) from December 1999 through June 2010. Most patients started with ETV since ETV was approved in Japan (July 2006). These patients were positive for serum HBsAg and HBV-DNA, and had elevated ALT at least twice before initiating treatment. We excluded patients who were co-infected with HCV or HIV, and those with liver cirrhosis or HCC. We discontinued NA treatment after obtaining verbal informed consent when patients satisfied the APASL stopping recommendation [12]; undetectable serum HBV-DNA by polymerase chain reaction (PCR) on three separate occasions at least 6 months apart in HBeAg-negative patients, and seroconversion from HBeAg-positive to HBeAb-positive and undetectable serum HBV-DNA by PCR for at least 12 months in HBeAg-positive patients. Treatment for at least 2 years was not a prerequisite for NA discontinuation in this study. Patients were followed up every one to three months to monitor blood chemistry and HBV markers. They received ultrasonography for the surveillance of HCC every 6 months. Relapse after NA discontinuation was defined as serum ALT elevation more than twice the upper limit of normal or serum HBV-DNA elevation higher than 5 log copy/mL at 2 consecutive examinations. Serum HBV-DNA levels were measured by COBAS TaqMan HBV test (Roche Molecular Systems, Inc., Pleasanton, CA). Serum HBsAg was quantitated by Architect assay (Abbott Japan, Co., Ltd., Tokyo, Japan). A part of serum samples were stored at −80°C until measurement. This study had been approved by Institutional Review Board for Clinical Research, Tokai University Hospital, and was performed in accordance with the Declaration of Helsinki.

### Statistical analysis

Pearson chi-square and Fisher exact probability tests were performed to compare the frequency distributions of categorical variables between groups. One-way analysis of variance was used to test the differences in means between groups for continuous variables. The probabilities of satisfying NA discontinuation criteria, relapse, disappearance of serum HBsAg, and progression to HCC were estimated using the Kaplan-Meier method, and differences between groups were compared using the log-rank test. Analyzed baseline factors included age, gender, HBV genotype, serum ALT, HBV-DNA, HBsAg, and liver histology assessed by METAVIR classification [14]. The factors with *P* value < .1 by univariate analysis were evaluated with a Cox proportional hazards regression model. All statistical analyses were performed using SPSS version 22 (SPSS Japan, Tokyo, Japan). All reported *P* values are 2-sided, with *P* < .05 considered statistically significant.

## Results

### Patient characteristics

The cohort consisted of 94 patients; 39 and 55 patients were negative and positive for HBeAg, respectively (Table 1). The HBeAg-negative patients were significantly older than the HBeAg-positive patients. Most patients were infected with genotype C HBV (84 % and 97 % in HBeAg-negative and -positive patients, respectively). The serum HBV-DNA levels were significantly higher in the HBeAg-positive patients. The mean ± SD of the follow-up period was 108 ± 42 (min-max: 18-182) and 111 ± 42 (min-max: 16-178) months in HBeAg-negative and -positive patients, respectively.

**Table 1:** Patient characteristics

Variables	HBeAg (-) (n=39)		HBeAg (+) (n=55)		*P* value
	no.	%	no.	%	
Age, <40/40-59/60≤	7/22/10	18/56/26	22/23/10	40/42/18	0.07
Age (y/o)	50.0 ± 11.8		44.3 ± 14.5		0.03
Gender, female/male	11/28	28/72	18/37	33/67	0.7
Drug, LAM/ETV	25/14	64/36	38/17	69/31	0.7
ALT (IU/L), <100/100-199/200≤	12/16/11	31/41/28	20/13/22	36/24/40	0.19
HBV-DNA (Log copy/mL), <6/6-7.6/7.6<	11/22/6	28/56/15	4/20/31	7/36/56	0.001
Genotype*, B/C	5/26	16/84	1/37	3/97	0.07
HBsAg (IU/mL)*, <1000/1000-10000/10000≤	7/11/5	30/48/22	3/9/8	15/45/40	0.09
Histology (METAVIR Staging)*, 1/2/3	13/8/6	48/30/22	11/13/7	35/42/23	0.6
Histology (METAVIR Grading)*, 1/2/3	7/17/3	26/63/11	8/15/8	26/48/26	0.3

*Data were not available in some patients

### Outcome of the patients

#### HBeAg-negative patients

**Fig. 1:**
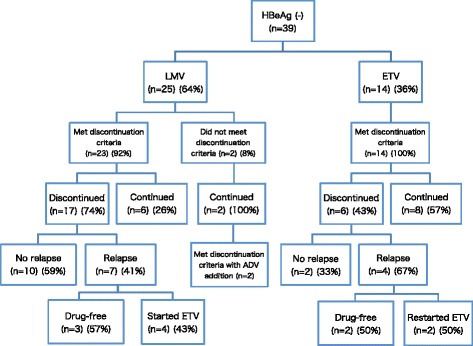
Flow chart of HBeAg-negative patients. LMV: lamivudine, ETV: entecavir, ADV: adefovir

Twenty-five (64 %) patients received LMV, while the remaining 14 (36 %) received ETV (Fig. 1). Most of the patients (23/25, 92 %) receiving LMV met the APASL stopping recommendation. Of 23 patients 17 agreed to stop LMV treatment, and 10 of these 17 patients (59 %) maintained remission. In 7 patients who relapsed, 3 achieved remission afterwards and 4 started ETV treatment. All of 14 patients (100 %) receiving ETV met the stopping criteria. Six of 14 patients agreed to stop ETV treatment, and 2 of 6 (33 %) maintained remission. In 4 relapsers, 2 achieved remission afterwards, and 2 restarted ETV. The mean ± SD of NA treatment duration until discontinuation was 40 ± 41 (min-max: 8–141) and 26 ± 17 (min-max: 9–50) months in patients who received LMV and ETV, respectively. The difference between LMV and ETV was not statistically significant. A total of 11 patients (48 %) experienced relapse. Most relapses occurred within 1 year after NA cessation. The cumulative relapse rate at 1 year, 2 years, and 5 years were 30 %, 35 %, and 39 %, respectively. In contrast, 44 % of patients were under control without NA administration.

#### HBeAg-positive patients

**Fig. 2:**
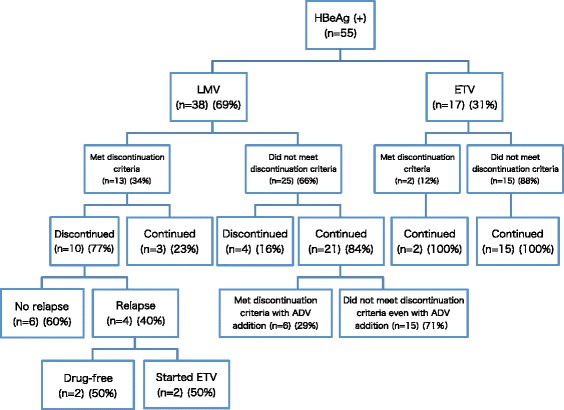
Flow chart of HBeAg-positive patients. LMV: lamivudine, ETV: entecavir, ADV: adefovir

Thirty-eight (69 %) patients received LMV and the remaining 17 (31 %) received ETV (Fig. 2). In patients who received LMV, 13 patients (34 %) met the stopping criteria. Six (60 %) of 10 patients who consented to stop LMV maintained remission. The mean ± SD of treatment duration until discontinuation was 44 ± 23 (min-max: 15–86) months. In 4 relapsers, 2 achieved remission afterwards, while 2 restarted ETV. Only 2 of 17 who received ETV met stopping criteria, and both continued ETV treatment. Finally, 4 patients (40 %) relapsed. The 1-year, 2-year, and 5-year cumulative relapse rates were 20 %, 30 %, and 30 %, respectively, whereas 15 % of patients were under control without NA administration.

#### Predictive factors for meeting the stopping criteria

**Fig. 3:**
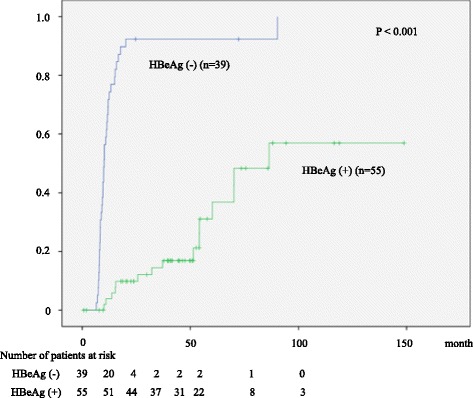
Cumulative rate of satisfying NA stopping criteria according to the HBeAg status

HBeAg-negative patients were more likely to meet the stopping criteria than HBeAg-positive patients (95 % vs. 27 %, *P* < 0.001, Fig. 3). We analyzed the predictive factors for meeting the stopping criteria in HBeAg-positive patients. Multivariate analysis revealed lower serum HBV-DNA level as the only predictive factor (*P* = 0.02). The proportion of patients who met the stopping criteria was 42 % (10/24) in those with serum HBV-DNA of 7.6 log copy/mL or less, whereas it was 15 % (5/31) in those with serum HBV-DNA higher than 7.6 log copy/mL.

#### Predictive factors for relapse

**Fig. 4:**
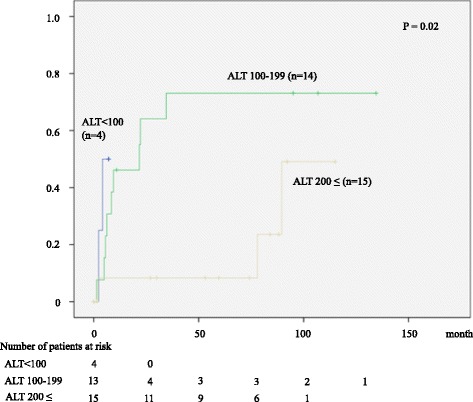
Cumulative rate of relapse after NA discontinuation according to the serum ALT levels

A total of 15 (45 %) among 33 patients who discontinued NA treatment relapsed, while the remaining 18 (55 %) maintained remission. We analyzed the baseline variables associated with relapse. Multivariate analysis demonstrated that serum ALT was the only predictive factor for relapse. Relapse rate was 50 % (2/4), 71 % (10/14), and 20 % (3/15) in patients with ALT < 100, 100–199, and ≥ 200 IU/mL, respectively. The patients with ALT ≥ 200 IU mL were more likely to maintain remission (Fig. 4, *P* = 0.02).

Serum ALT elevated up to more than 500 IU/mL in 4 of 15 relapsers (27 %), however, none experienced decompensation.

#### HBsAg disappearance

**Fig. 5:**
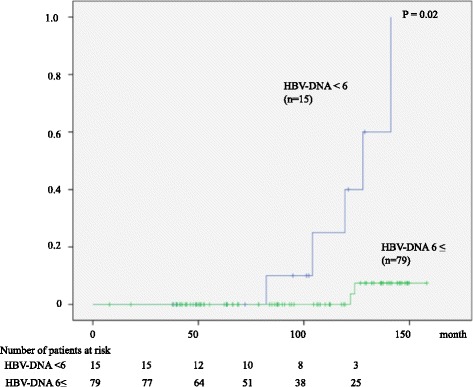
Cumulative rate of HBsAg loss according to the viral load

Serum HBsAg disappeared in 7 patients (7.4 %), resulting in an annual HBsAg disappearance rate of 0.8 %; 1.4 % and 0.4 % for HBeAg-negative and -positive patients, respectively. We analyzed the baseline factors associated with HBsAg loss. Serum HBV-DNA was the only predictive variable by multivariate analysis; HBsAg loss was observed in 5 of 15 patients (33 %) with serum HBV-DNA less than 6 log copy/mL, while 2 of 79 patients (3 %) with serum HBV-DNA of 6 log copy/mL or higher (*P* = 0.001). The accumulated HBsAg loss was observed more frequently in those with lower HBV-DNA (Fig. 5, *P* = 0.02).

#### HCC progression

**Fig. 6:**
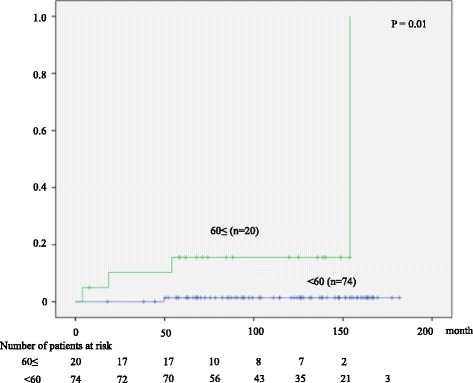
Cumulative rate of HCC occurrence according to the age

Five of 94 (5.3 %) patients progressed to HCC, resulting in an annual HCC progression rate of 0.6 % in both HBeAg-negative and -positive patients. Age was the only predictive variable for HCC progression by multivariate analysis (*P* = 0.01). Only one of 74 (1.4 %) patients younger than 60 years progressed to HCC, whereas did 4 of 20 (20 %) patients aged 60 or older (*P* = 0.01, Fig. 6). Four patients died, all of whom were HBeAg-positive. One died of HCC and the remaining 3 died of the diseases unrelated to the liver (pancreatic cancer, gastric cancer, and unknown).

## Discussion

In approximately 9 years after initiating NA administration, 44 % (17/39) of HBeAg-negative patients and 15 % (8/55) of HBeAg-positive patients were under control without NA. In other words, more than half of HBeAg-negative and most of HBeAg-positive patients still need NA therapy, suggesting difficulty in stopping NA therapy.

In this study we adopted the APASL stopping recommendation [12]. Most of the HBeAg-negative patients (95 %) satisfied this stopping criteria, whereas only one forth of the HBeAg-positive patients (27 %) did. These results are in agreement with a study [15] reporting that seroconversion from HBeAg-positive to HBeAb-positive occurred only in 38 % of HBeAg-positive patients through 4 year-treatment of ETV. Multivariate analysis revealed that patients with lower serum HBV-DNA levels were more likely to meet the stopping criteria in concordance with the previous study [15]. Thus, patients with higher serum HBV-DNA will have difficulty to cease NA treatment once it started.

In HBeAg-negative patients, the cumulative relapse rate after discontinuation of NA therapy was 48 % (11/23), which was comparable with the previous studies [9, 16-19]. Jeng et al. [19] evaluated off-therapy durability in 95 patients who discontinued ETV treatment according to the APASL stopping recommendation as we adopted. The cumulative 1-year relapse rate was 45.3 %. On the other hand, another prospectively study [20] enrolling 184 patients in which ETV was stopped according to the APASL stopping recommendation demonstrated an exceptionally high cumulative 1-year relapse rate of 91.4 %. Although reason for the discrepancy in the reported relapse rates is unclear, the differences in patients background and study design may be involved. The AASLD and EASL recommend long-term NA therapy until HBsAg seroclearance is achieved, however, this goal is unrealistic considering very low HBsAg seroclearance rate (<1 % per year [10]). The others and we showed that approximately half of the HBeAg-negative patients remained remission after cessation of NA therapy according to the APASL stopping recommendation. Thus, NA therapy can be stopped with proper monitoring.

In the HBeAg-positive patients, cumulative relapse rate after discontinuation of NA therapy was 40 % (4/10), which is in concordance with other studies ranging 29–77 % [5, 6, 8, 20-24]. According to a retrospective study of Song et al. analyzing 98 HBeAg-positive patients who stopped LMV therapy after HBeAg seroconversion, the cumulative relapse rates at 1 year and 2 years were 37.5 % and 49.2 %, respectively [5].

Our study demonstrated that relapse was less frequently observed in patients with higher ALT levels in agreement with a previous study [6]. Patients with high ALT indicating strong immunological reaction against HBV may be on the transition state from immune clearance phase to low replicative phase in which relapse is relatively rare. Although lower baseline serum HBV-DNA [5, 6, 19] and younger age [9, 25–27], and longer treatment duration [5, 27] were associated with lower relapse rate in other studies, we could not find these associations.

Four of 15 relapsers (27 %) underwent serum ALT flare above 500 IU/L, however, they did not progress to liver failure. ALT flare with 10 times higher than normal upper limit was reported in up to 20 % [2, 3] of patients who discontinued NA treatment. We similarly observed ALT flare, but did not experience decompensation as previous studies [9, 19, 20, 22].

HBsAg disappeared at an annual rate of 0.8 %; 1.4 % in HBeAg-negative and 0.4 % in HBeAg-positive patients. These results are compatible with other studies [28-32]. Low baseline serum HBV-DNA was the only predictor for HBsAg disappearance in our study, in agreement with a previous study [33]. Kim et al. [33] revealed 0.33 % annual HBsAg seroclearance rate by 6-year-follow-up of 5409 patients (two-thirds were HBeAg positive) receiving LMV or ETV therapy, and demonstrated baseline low HBV-DNA, high ALT, HBeAg negativity, and absence of cirrhosis as predictors for HBsAg loss.

The progression to HCC was observed at an annual rate of 0.6 % in both HBeAg-negative and -positive patients, which results are in agreement with previous studies [33-36]. The decrease of HCC occurrence by long-term NA treatment is confirmed by a randomized study [1] and a meta-analysis [37]. The only predictive factor for HCC progression was age of 60 years or older, which is also reported in a previous study [36].

This study has limitations; the study was conducted in a retrospective manner in a single institution, and number of patients was small. Our patients predominantly consisted of genotype C. However, the data on off-treatment durability of NA’s antiviral effect are still insufficient, and we believe our results provide important information.

## Conclusions

Approximately half of the patients did not relapse after cessation of NA therapy in both HBeAg-negative and -positive patients. The relapsers did not experience decompensation. Therefore, NA therapy could be discontinued with close monitoring if the APASL stopping recommendation is satisfied even in HBeAg-negative patients.
